# Secular trends of salted fish consumption and nasopharyngeal carcinoma: a multi-jurisdiction ecological study in 8 regions from 3 continents

**DOI:** 10.1186/1471-2407-13-298

**Published:** 2013-06-19

**Authors:** Hiu-Ying Lau, Chit-Ming Leung, Yap-Hang Chan, Anne Wing-Mui Lee, Dora Lai-Wan Kwong, Maria Li Lung, Tai-Hing Lam

**Affiliations:** 1 School of Public Health, University of Hong Kong, Hong Kong, China; 2 Department of Clinical Oncology, Queen Mary Hospital, University of Hong Kong, Hong Kong, China; 3 Department of Clinical Oncology, Pamela Youde Nethersole Eastern Hospital, Chai Wan, Hong Kong; 4 Epidemiology Group, Center for Nasopharyngeal Carcinoma Research, Hong Kong RGC Area of Excellence Scheme, Hong Kong, China; 5 School of Public Health, The University of Hong Kong, 21 Sassoon Road, Hong Kong, China

**Keywords:** Nasopharyngeal carcinoma, Salted fish consumption, Tobacco, Secular trend, Ecological study

## Abstract

**Background:**

Despite salted fish being a classical risk factor of Nasopharyngeal Carcinoma (NPC), whether secular trends in salted fish consumption worldwide accounted for changes in NPC rates were unknown. The relationship between vegetable and cigarette consumption to NPC risk worldwide were also largely uncertain. We investigated the longitudinal trends in standardised NPC incidence/mortality rates across 8 regions and their associations with secular trends in salted fish, vegetable and tobacco consumptions.

**Methods:**

Age standardised mortality rate (ASMR) and age standardised incidence rate (ASIR) of NPC were obtained from the WHO cancer mortality database and Hong Kong Cancer Registry. Per capita consumption of salted fish, tobacco and vegetables in Hong Kong and 7 countries (China, Finland, Japan, Portugal, Singapore, United Kingdom and United States) were obtained from the Food and Agriculture Organization of the United Nation (FAO) and Hong Kong Trade and Census Statistics. Pearson correlation and multivariate analysis were performed to examine both crude and adjusted associations.

**Results:**

There were markedly decreasing trends of NPC ASIR and ASMR in Hong Kong over the past three decades, which were correlated with corresponding secular changes in salted fish consumption per capita (Pearson r for 10 cumulative years : ASIR = 0.729 (male), 0.674 (female); ASMR = 0.943 (male), 0.622 (female), all *p < 0.05 except for female ASMR*). However such associations no longer correlated with adjustments for decreasing tobacco and increasing vegetable consumption per capita (Pearson r for 10 cumulative years: ASIR = 2.007 (male), 0.339 (female), ASMR = 0.289 (male), 1.992 (female), all *p > 0.05*). However, there were no clear or consistent patterns in relations between NPC ASIR and ASMR with salted fish consumption across 7 regions in 3 continents.

**Conclusions:**

Our results do not support the notion that changes in salted fish consumption had played an important role in explaining secular trends of NPC rates in Hong Kong and worldwide. Further studies should explore other lifestyle and genetic factors. However, our findings do support the potentially protective effects of vegetable consumption against NPC.

## Background

The distribution of nasopharyngeal carcinoma (NPC) has a distinctive geographic variation. Being a rare malignancy worldwide, it has particularly high rates in Southern China [1]. Residual risk remained high among migrants of Chinese descent [2] to non-endemic areas, with intergenerational decline [3], suggesting that the aetiology of NPC is a complex interplay between genetic influences and environmental exposures distinct in these populations.

Among known environmental exposures relevant to the aetiology of NPC; dietary consumption of salted fish is the most well recognised risk factor. First suggested by Ho and colleagues [4] to have a link with the high incidence of NPC in Southern China Guangdong Province in the early 1970s, subsequent studies quite consistently reported positive associations between salted fish consumption and risk of NPC in Chinese [5] and other ethnicities [6] such as Thai and Tunisian [7], and that early exposures during the weaning period in childhood may play a critical role [1,8]. What makes salted fish an intriguing item to look at is that nitrosamine in Cantonese style salted fish is carcinogenic to humans and this food item is rated as a group 1 carcinogen by the International Agency for Research on Cancer (IARC)[9], whereas other salted fish are only classified as group 3 carcinogen [9]. This can be explained by the different levels of nitrosamines in various types of salted fish, depending on the method of preservation [10]. However, the low disease rates of NPC even in endemic regions render case-control studies to be the only feasible epidemiological method, of which limitations and deficiencies are well-recognised. The extent to which notorious effects of recall bias [11], lack of adjustment of caloric intake [12] and other confounders may affect the validity of the study findings remains unknown. Whether any publication bias may exist due to active reporting of salted fish consumptions as a classical risk factor of NPC is also uncertain. Although Cantonese-style salted fish has been classified by IARC as a group 1 carcinogen [9], the putative main biological active component N-nitrosodimethylamine, is only classified as a group 2A carcinogen [13]. Such incoherence makes the aetiological role of salted fish especially worthwhile for more thorough re-exploration.

In the past 30 to 40 years, the incidence and mortality rates of NPC have dropped noticeably in several Asia regions particularly among some Chinese subpopulations [14,15]. Given the wide range of the relative risk of high salted fish consumption ranging from 2.5–17.2 [16],which translates into population attributable risk (PAR) of 39%–78%, adopting exposure prevalence of salted fish consumption in the population, it would be of interest to investigate whether the secular trend of NPC disease rates is explained by a corresponding change in salted fish consumption in relevant jurisdictions. To our best knowledge, reports of such studies were not available.

We, therefore, carried out this Hong Kong and multi-site ecological study to investigate the relationship between secular trends of salted fish consumption and standardised NPC incidence and mortality rates over 30 years. We also included an analysis in Hong Kong taking into account other key lifestyle related factors, including tobacco smoking [17] and vegetable [18] consumption, which were reported to be a risk and protective factor of NPC respectively.

## Methods

### Study sites

In this ecological study, we focused on Hong Kong, an endemic area of NPC, and 7 countries in 3 continents including: China, Finland, Japan, Portugal, Singapore, United Kingdom and United States. Hong Kong was chosen due to its high prevalence of NPC, with the advantage of a comprehensive cancer statistics database and the availability of relevant risk/protective factor profiles. China was also selected for investigation, since there is a substantial variation in NPC risk of Chinese who lived in different parts of China. For example those who live in the Northern parts of China have a lower incidence rate than people who live in Southern China [15]. Another area with a relatively high NPC mortality rate is Singapore, which attracted much attention in previous NPC case control studies [19,20]. For low risk areas, Japan, Finland, Portugal, United Kingdom and United States were selected because of their rather complete cancer statistics.

### NPC incidence and mortality statistics

NPC age-standardised mortality rates (ASMR) and age-standardised incidence rates (ASIR) were obtained from various sources. NPC age-standardised incidence and mortality rates in Hong Kong during 1978 to 2008 were derived from Hong Kong Cancer Registry [21]with the world 1966 standard population as reference. NPC mortality statistics of other jurisdictions were collected from the WHO cancer mortality database [22-24], and age-standardised rates were also adjusted to the world standard population 1966. The code of cancer site followed the International Classification of Disease (ICD) coding system, with NPC coded as 147 (ICD-8, WHO 1967; ICD-9, WHO 1977) and C11 (ICD-10, WHO 1992).

### Salted fish consumption per capita

#### Hong Kong SAR trade statistics and census

Salted fish consumption in Hong Kong was estimated from import, export and re-export volumes of dried fish, and whether or not salted but not smoked provided by the Service Centre on Trade Statistics, Census and Statistics Department, Hong Kong SAR Government. The database contains monthly and annual tallies of quantity, value and import by country of origin (of the product), import by country of consignment (origin of shipment), country of export, and country of re-export according to Harmonized Commodity Description and Coding System of the World Customs Organization. Domestic consumption was calculated from the difference between exports, re-exports and imports. All these data were acquired from the archive database of Census and Statistics Department. Salted fish consumption per capita for each year was then estimated according to the formula: (imports − exports − re-exports) ÷ mid-year population in Hong Kong retrieved from Population Census and By-census, Demographic Statistics Section, Census and Statistics Department, 1978–2008[25] .

#### Food and Agriculture Organisation (FAO), United Nations

Worldwide salted fish consumptions were compiled by productions, imports, exports and re-exports on fish, dried, salted or smoked from Fishery and Aquaculture Statistics Database, FAO [26], United Nations. This database contains statistics on the annual production, imports, exports and re-exports of fishery commodities by country and commodities in terms of volume and value from 1976. For every country, per capita consumption was calculated from the difference between exports, re-exports, imports and production, i.e. [(import + production) – export– re-export] ÷ total population of the defined jurisdiction in each year. Total population is estimated from FAOSTAT Population module [27][26].

### Tobacco consumption per capita

Per capita cigarette consumption in Hong Kong was estimated by Hong Kong Council on Smoking and Health (COSH) via General Household Survey and Thematic Household Survey 2008[28] in reporting average daily cigarette consumption among smokers(sticks), number of smokers and percentage of smokers in Hong Kong from 1982–2008. Per capita consumption was calculated by: Daily average consumption × No. of daily smokers (aged 15 and over) ÷ Population (all age) × 365 days = Per capita consumption (stick/capita/year). Statistic on tobacco tax was also collected from COSH. Data on percentage increase in tobacco tax was plotted with per capita consumption of tobacco.

### Vegetable consumption per capita

Per capita fresh vegetable consumption data in Hong Kong from 1991 to 2009 were obtained from the Department Annual Report by Agriculture, Fisheries and Conservation Department HKSAR [29]. All per capita calculations in Hong Kong were divided by mid-year population obtained from Population Census and By-census 1991–2009, Demographic Statistics Section, Census and Statistics Department [25].

### Statistical analysis

NPC incidence and mortality rates were estimated from 1978–2008, as cases or deaths per 100,000 persons. The rates were age-standardised to the world 1966 standard population for all localities using 18 age groups (<1, 1–4, 5–9,…, 80–84, 85+). Age-standardised mortality/incidence and male/female ratio in age-standardised incidence and mortality rates were also calculated.

Non-linear regression of least square method was used for fitting any incomplete data point from 1978–2008. Pearson correlation analysis was used to examine the relations between cumulative consumption of salted fish, vegetable and tobacco in relation to ASIR and ASMR from 1991–2008 in Hong Kong. Multivariable linear regression analysis was performed to adjust for potential confounding effects of tobacco and vegetable consumption during the period of 1991–2008. Repeated analysis using various duration of cumulative salted fish intake prior to the NPC disease rate outcomes was performed to test the robustness of estimates based on differential assumptions in the latency period of cancer development subsequent to relevant exposures (range: 0, 5, 10 years). A *p* value smaller than *0.05* (two-sided) was considered statistically significant. All the data was analysed by SPSS Statistics 17.0.

## Results

### Age-standardised incidence and mortality rates in Hong Kong from 1978–2008

**Figure 1:**
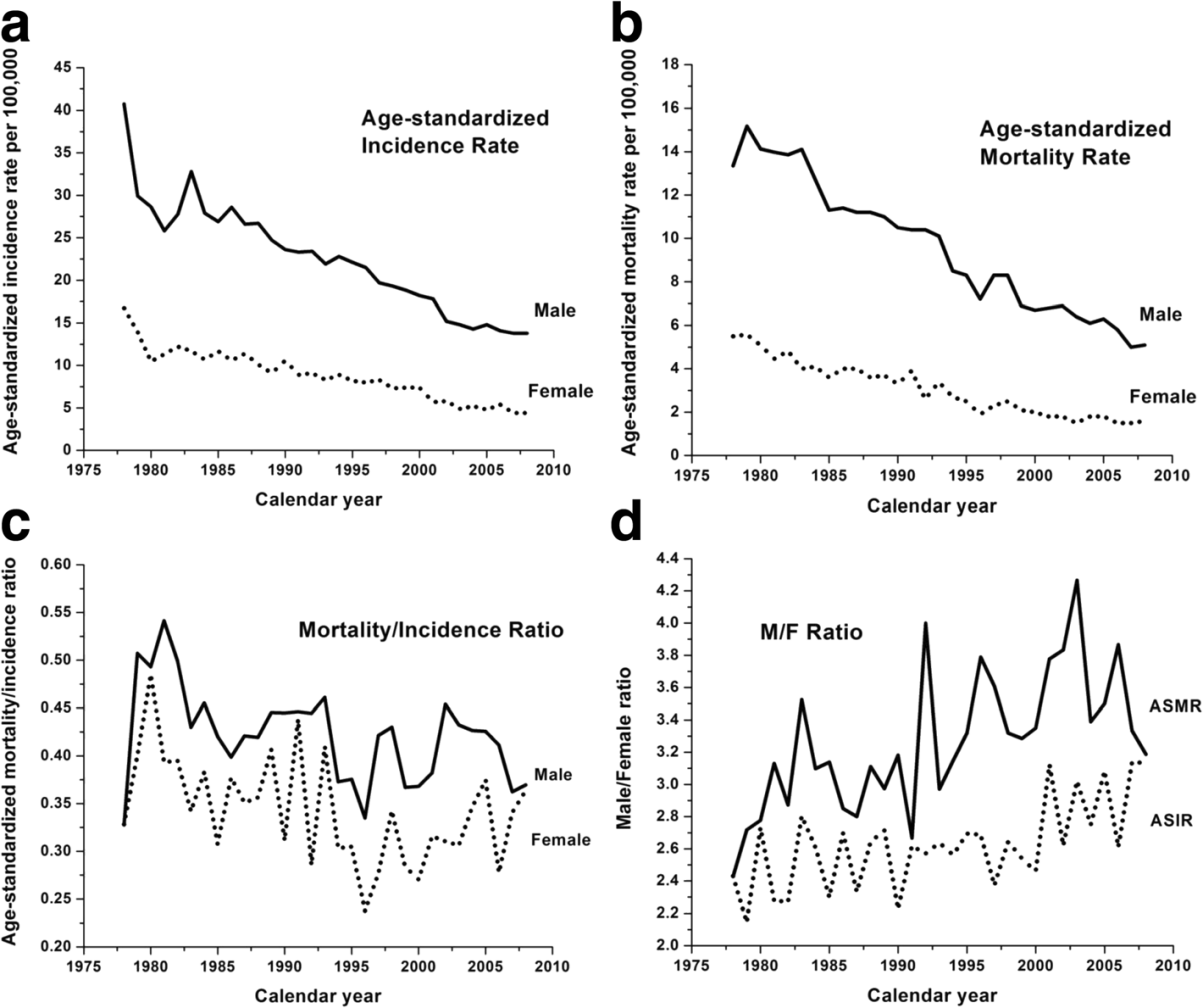
**Cancer statistics for NPC in Hong Kong from 1978 to 2008.** (**a**) Age-standardized incidence rate; (**b**) Age-standardized mortality rate; (**c**) Age-standardized mortality/incidence ratio; (**d**) Male/Female ratio in age-standardized incidence and mortality rates.

As shown in Figure 1a and b, both ASIR and ASMR for males and females showed decreasing trends over the last 30 years, with males having consistently higher rates than females. By 2008, ASIR and ASMR for males had decreased by 66.1% and 61.7%, respectively, compared to the late 1970s. Similarly, ASIR and ASMR for females had also decreased by 73.7% and 70.9%, respectively.

The age standardised mortality/incidence ratio (Figure 1c) for both genders, albeit with notable fluctuations, followed an overall slowly declining trend over the past 30 years with estimates for males being consistently higher than females. The male/female ratios for ASIR and ASMR, as presented in Figure 1d, maintained a value of around 3 over the past 30 years with a slowly rising trend. The ASMR male/female ratio was higher than that for ASIR throughout the whole period.

### Secular trend of salted fish consumption estimates in Hong Kong from 1978–2008

**Figure 2:**
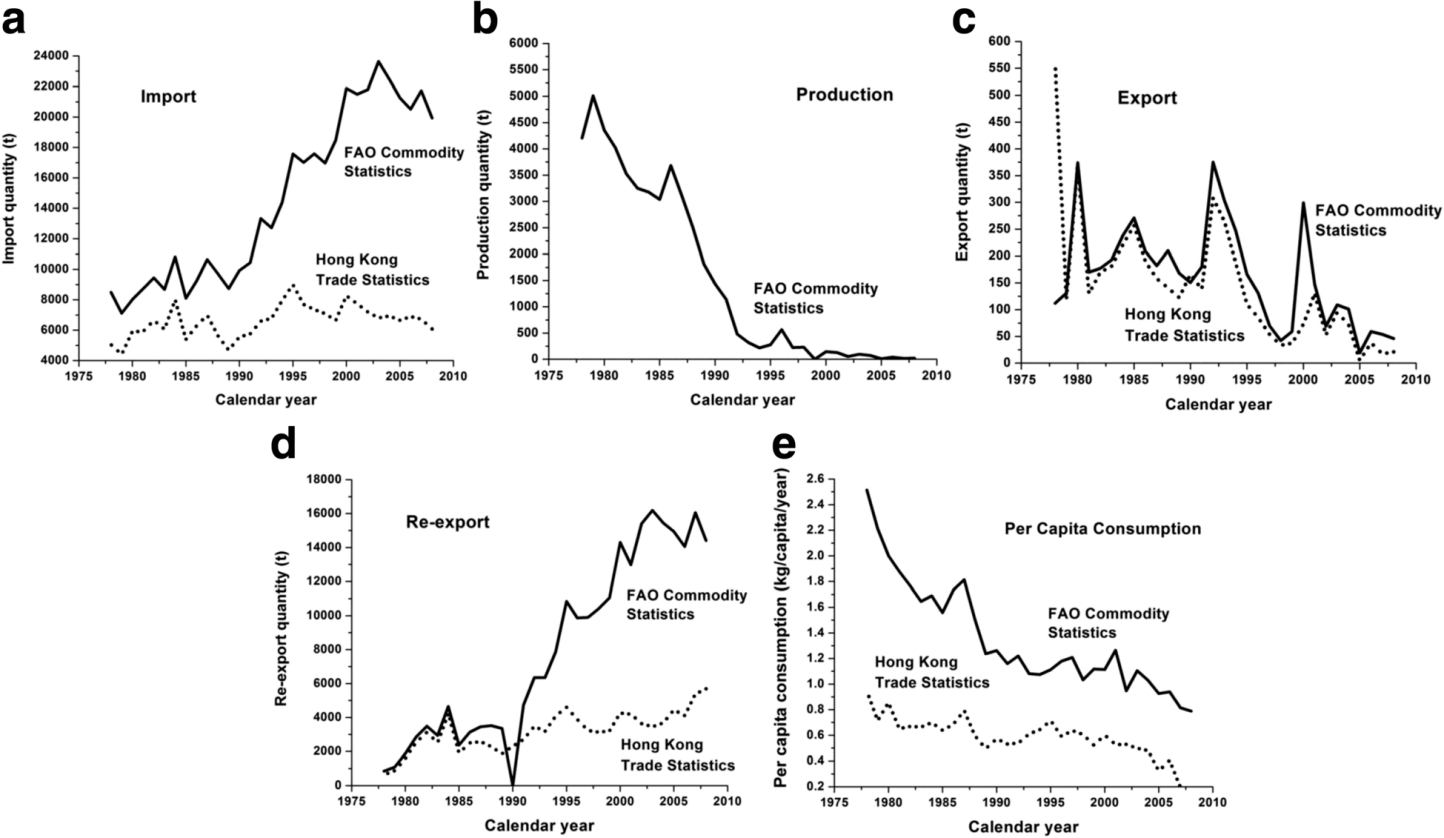
**FAO commodity statistics of fish, dried, salted or smoked (solid line), and Hong Kong trade statistics on dried fish, whether or not salted but not smoked (dotted line), 1978–2008.** (**a**) Import; (**b**) Production; (**c**) Export; (**d**) Re-export; (**e**) Per capita consumption. Production data are not available at Hong Kong trade statistics. Pearson correlation on per capita consumption estimated by FAO commodity statistics and Hong Kong trade statistics is 0.780 (p < 0.01).

The salted fish consumption based on the FAO and Hong Kong Trade Statistics is presented in Figure 2a-e. The import data (Figure 2a) from separate databases gave distinct trends. FAO statistics showed more markedly increased imports over 30 years, while Hong Kong Trade Statistics increased steadily from 1978 to 2008. Only FAO data was available for production of salted fish (Figure 2b) which decreased sharply from the 1980s to the mid-1990s such that by 1999 onwards it had declined to around zero. Apart from the disparities at the beginning of the curves, both sources produced similar trends in terms of export quantity (Figure 2c). Annual export was reduced from the peak of 374 and 559 ton in the 1980s for FAO and Hong Kong Trade Statistics, respectively, to 46 and 21 tons in late 2000s. Consistent with the higher estimates of salted fish imports from the FAO statistics than the Hong Kong Trade Statistics, the former also showed a corresponding significant increase in re-exports of salted fish from Hong Kong (Figure 2d). Based on these raw data and the formula defined *a priori*, per capita consumption was derived (Figure 2e). Despite notable differences in some of the raw parameter estimates, the overall salted fish consumption per capita derived from the FAO and the Hong Kong Trade Statistics was strongly correlated (Pearson correlation r = 0.780, *p < 0.05*) and showed a consistently progressive decline.

### Relation between salted fish consumption and NPC in Hong Kong

Table 1 shows that Pearson correlation coefficient (r) was consistently strong between salted fish consumption per capita and both ASIR (Male: Range 0.700 to 0.729, all *p < 0.05*; Female: Range 0.668 to 0.704, all *P < 0.05*) and ASMR of NPC (Male: Range 0.627 to 0.943, all *p < 0.05*; Female: cumulative consumption of 5 years = 0.611, *p < 0.05*). Repeated analyses using the Hong Kong Trade Statistics data (Table 1b) yielded similar results compared to the FAO statistics (Table 1a). No clear relationship for the number of cumulative years and Pearson’s r was observed.

**Table 1:** Correlation coefficient (r) from Pearson correlation analysis between cumulative salted fish consumption per capita (per kg) and age-standardized incidence and mortality rates in Hong Kong, 1991–2008

	**(a) FAO commodity statistics**			
**Year of cumulative salted fish consumption per capita**	**Age-standardized incidence rate**		**Age-standardized mortality rates**	
	**Male**	**Female**	**Male**	**Female**
0	0.700*	0.668*	0.627*	0.430
5	0.710*	0.704*	0.789*	0.611*
10	0.729*	0.674*	0.943*	0.622
	**(b) Hong Kong trade statistics**			
**Year of cumulative salted fish consumption per capita**	**Age-standardized incidence rate**		**Age-standardized mortality rates**	
	**Male**	**Female**	**Male**	**Female**
0	0.709*	0.762*	0.652*	0.502*
5	0.792*	0.830*	0.882*	0.729*
10	0.471	0.477	0.893*	0.395

* Significant at 0.05 level.

The cumulative salted fish consumption up to the specified time point was estimated by aggregating annual salted fish consumption per capita estimates in all years prior to that year up to 1991. (a) Cumulative salted fish consumption per capita was estimated from FAO commodity statistics on fish, dried, salted or smoked. (b) Cumulative salted fish consumption per capita was estimated from Hong Kong trade statistics on dried fish, whether or not salted but not smoked.

### Tobacco consumption and relations with NPC in Hong Kong

**Figure 3:**
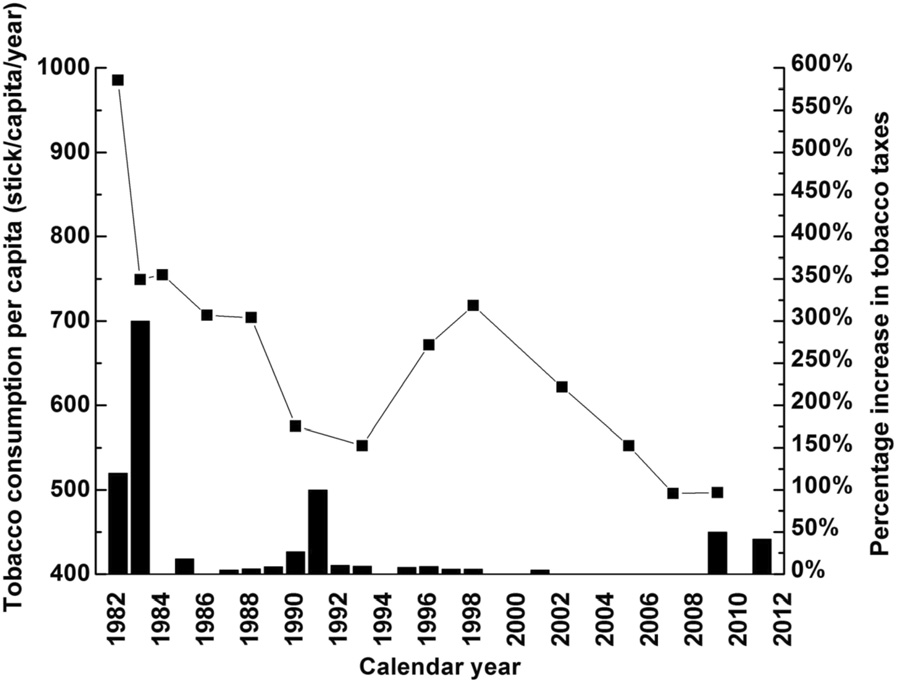
Per capita cigarette consumption (line + symbol) versus tobacco tax increase (column) in Hong Kong, 1982–2009.

Figure 3 showed annual tobacco consumption had an overall decreasing trend over the past 3 decades, from 986 sticks per capita in 1982 to 497 sticks per capita in 2009 (about 50% decrease), attributable to successful and comprehensive tobacco control measures, including taxation which might account for the observed marked drop of tobacco consumption in 1983 and the early 1990s (respectively 300% in 1982 and 100% increase in 1991 in tobacco tax). Table 2 shows correlation between cumulative cigarette consumption per capita and both ASIR (Male: Range 0.646 to 0.694, all *p < 0.05*; Female: Range 0.685 to 0.735, all *p < 0.05*) and ASMR of NPC (Male: Range 0.598 to 0.941, all *p < 0.05*; Female: cumulative 5 years = 0.592, *p < 0.05*).

**Table 2:** Correlation coefficient (r) from Pearson correlation analysis between cumulative cigarette consumption per capita (per stick) and age-standardized incidence and mortality rates in Hong Kong, 1991–2008

**Year of cumulative cigarette consumption per capita**	**Age-standardized incidence rate**		**Age-standardized mortality rates**	
	**Male**	**Female**	**Male**	**Female**
0	0.694*	0.735*	0.598*	0.446
5	0.646*	0.685*	0.768*	0.592*
10	0.635	0.625	0.941*	0.563

* Significant at 0.05 level.

### Vegetable consumption and relations with NPC in Hong Kong

**Figure 4:**
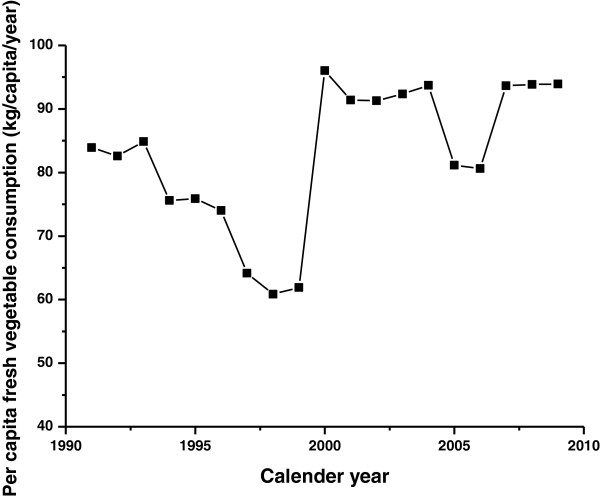
Fresh vegetable consumption trend in Hong Kong, 1991–2009.

With an overall increasing trend over the past 2 decades, the annual per capita consumption of vegetables increased from 85 kg per capita in 1991 to 94 kg per capita in 2009 (Figure 4). Pearson r increased with increasing number of cumulative years (Table 3). A negative correlation suggested vegetable consumption is a strong protective factor for NPC. Pearson correlation for ASIR ranged from −0.721 to −0.771 in males and −0.528 to −0.766 in females, whereas ASMR in males ranged from −0.657 to −0.940 and in females cumulative consumption of 5 years was −0.661. Pearson’s r stayed similar as the cumulative years increased.

**Table 3:** Correlation coefficient (r) from Pearson correlation analysis between cumulative fresh vegetable consumption per capita (per kg) and age-standardized incidence and mortality rates in Hong Kong,1991–2008

**Year of cumulative fresh vegetable consumption per capita**	**Age-standardized incidence rate**		**Age-standardized mortality rates**	
	**Male**	**Female**	**Male**	**Female**
0	−0.466	−0.528*	−0.364	−0.294
5	−0.721*	−0.752*	−0.657*	−0.661*
10	−0.771*	−0.766*	−0.940*	−0.599

* Significant at 0.05 level.

### Multivariate model

Table 4 shows that in the multivariable model, associations between per capita consumption of salted fish and ASIR/ASMR of NPC were no longer statistically significant except ASIR in males at 5 cumulative year level (ASIR: β = 2.744, *p < 0.05*). Tobacco consumption showed significant associations with male ASIR at 5 (β = −2.694, *p < 0.05*) and 10 cumulative years (β = −3.142, *p < 0.05*). ASIR in males and females both showed significant association for vegetable consumption (ASIR in 5 cumulative years: β: −0.917 in males; −0.794 in females; cumulative 10 years: β: −1.793 in males; −2.408 in females; all *p < 0.05*). Repeated analysis using data from Hong Kong Trade Statistic for salted fish consumption gave similar results (data not shown).

**Table 4:** Beta (β) regression coefficient from multivariate regression analysis of NPC age-standardized incidence and mortality rates on cumulative salted fish (per kg), cigarette (per stick) and fresh vegetable consumption (per kg) in Hong Kong, 1991–2008

**Cumulative window (Years)**	**Independent variable**	**Age-standardized incidence rate**		**Age-standardized mortality rates**	
		**Male**	**Female**	**Male**	**Female**
0	Salted fish	0.473	0.345	0.463	0.238
	Smoking	0.195	0.321	0.145	0.189
	Fresh-vegetables	−0.213	−0.249	−0.140	−0.119
5	Salted fish	2.744*	1.740	1.324	1.607
	Smoking	−2.694*	−1.589	−0.814	−1.501
	Fresh-vegetables	−0.917*	−0.794*	−0.402	−0.725
10	Salted fish	2.077	0.339	0.289	1.992
	Smoking	−3.142*	−2.038	0.306	−1.571
	Fresh-vegetables	−1.793*	−2.408*	−0.363	−0.252

* Significant at 0.05 level.

Cumulative salted fish consumption per capita was estimated from FAO commodity statistics on fish, dried, salted or smoked.

### Multi-jurisdiction ecological analysis

**Figure 5:**
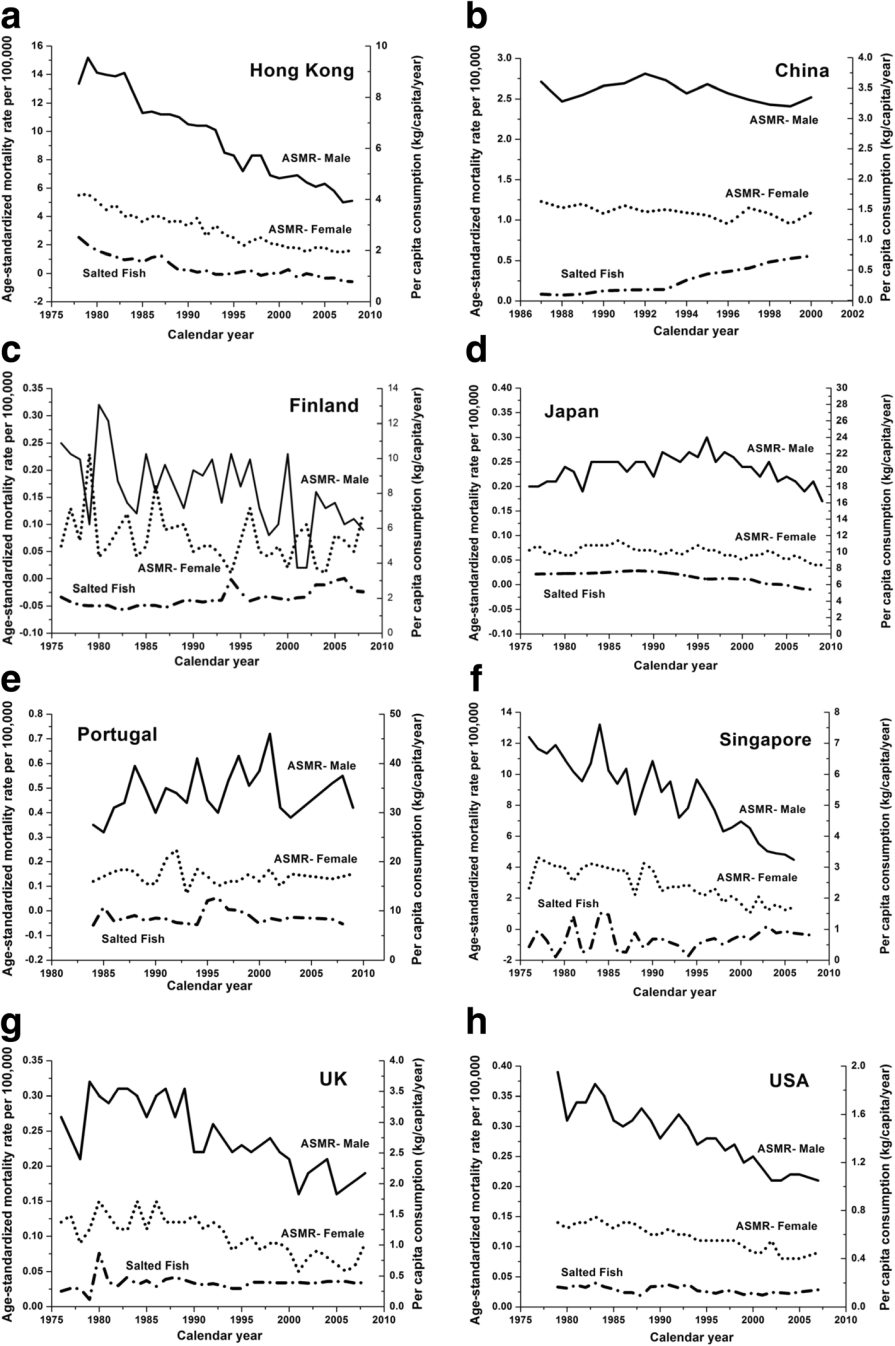
**NPC Age-standardized mortality rates in males (solid line) and females (dotted line) versus salted fish consumption per capita (dash-dotted line) in multiple jurisdictions.** (**a**) Hong Kong; (**b**) China (**c**) Finland; (**d**) Japan; (**e**) Portugal; (**f**) Singapore; (**g**) United Kingdom and (**h**) United States.

Marked reduction in NPC ASMR for both genders was observed in Hong Kong (Figure 5a) over the past three decades, with a corresponding drop in salted fish consumption. However the link between salted fish consumption and NPC ASMR was not observed in other jurisdictions. In China (Figure 5b), salted fish consumption increased from the early 1990s up to 2000. Both ASMR for males and females stayed rather stable over the years, with a higher ASMR in males which fluctuated at around 2.5 per 100,000 person years while ASMR in females was around 1.1 per 100,000 person years. Figure 5c shows the ASMR trend in Finland from 1976 to 2008. Males had a slightly higher mortality rate than females and the ASMR range was narrow. Japan (Figure 5d) had stable ASMRs for both males and females over the past three decades. In Portugal, (Figure 5e) no clear trend was seen, although a slight drop in male NPC death rates was observed. ASMR in males in Singapore (Figure 5f) showed a large reduction through the whole period while the ASMR in females only dropped slightly. Consumption of salted fish had not changed much during this period. In the United Kingdom (Figure 5g), there was an overall 20% decrease in ASMR among males from early 1980s to late 2000s. In females the drop was smaller and salted fish intake did not change. The United States (Figure 5h) had a very similar trend to UK, but a significant trend was seen only for ASMR in males.

## Discussion

This is the first multi-jurisdiction ecological study that examined the associations between secular trends of NPC risk and salted fish consumption per capita. We also adjusted for the potentially confounding effects of two most important lifestyle factors, namely tobacco and vegetable consumption, using per capita estimates. We included 7 regions in 3 continents to include both endemic and low-risk areas, to analyse the pattern with a more global perspective.

We observed a decline in both ASIR and ASMR of NPC in Hong Kong over the past three decades. Higher NPC ASMR was observed in males than females in most of the jurisdictions included, consistent with previous studies [11,30,31], which may be due to a combination of differential risk factor exposures such as greater tobacco consumption in males and genetic factors. The rather consistent; more than 3-fold risk in males compared with females needs further investigation. Our results showed markedly decreasing trends in salted fish intake and tobacco consumption, but an increasing trend in vegetable consumption in Hong Kong over the past several decades. All of these environmental risk factors were individually highly correlated with ASMR and ASIR. The decrease in salted fish consumption may represent a change in dietary culture with westernisation of dietary patterns among Hong Kong citizens from a traditional Cantonese diet and from increased awareness of the carcinogenicity of salted fish. Decrease in intake of salted fish should lower the risk of an individual getting NPC if the relationship is causal [5].

There were no substantial changes in the proportion of Cantonese residing in Hong Kong, thus any changes noted should not be confounded by migration effect. Additional file 1: Tables S1-S3 show the population structure in Hong Kong from 1961–2006. Nevertheless, in the multivariable model, the relations between salted fish consumption and NPC ASIR and ASMR were attenuated and became mostly insignificant. In contrast, the negative association between vegetable consumption and NPC ASIR remained statistically significant after adjustment and was robust across varying estimates of cumulative exposure windows prior to NPC outcomes. Consistent with findings from previous case-control studies [18,32], dietary intake of fruits and vegetables may have a protective effect against NPC due to its antioxidant contents, potentially interfering with the actions of nitrosamines [33]. World Cancer Research Fund (WCRF) panel suggested fruit and vegetable consumption have limited to suggestive levels of evidence for protection against NPC [34], which is consistent with the result found in this study. Although positive association was observed for cigarette consumption and NPC ASIR in males, previous literature suggested that for lung cancer, of which smoking is known as one of the major risk factor, the lag year for the disease from exposure to cancer onset was 25–30 years [35], it is expected that the lag for NPC should not be shorter if not similar to lung cancer. As our study is ecological, further analytical studies, such as case control studies with genetic, viral and other biomarkers are warranted to examine the link between salted fish and NPC in Hong Kong, taking into account smoking, vegetable consumption and other potential confounders.

Despite the parallel secular trends of salted fish consumption and NPC rates in Hong Kong, the same pattern was not observed in all other jurisdictions. For instance, in China, salted fish consumption increased over the years and there was no sign in apparent increase of ASMR in both males and females. Similarly, there are jurisdictions where NPC ASIR/ASMR had markedly decreased, but with no accompanying drop in salted fish consumption. Caution needs to be taken in interpretation, since the salted fish consumed in other countries might not be the Cantonese-styled salted fish, which is categorised as a group I carcinogen by IARC. The effect of salted fish on NPC development might only have a notable impact among Hong Kong Chinese but not in other populations, due to the higher underlying disease rates of NPC in Hong Kong, or could be due to genetic differences e.g. nitrosamine metabolism [36]. Indeed, the vast majority of previous case-control studies on salted fish consumption and NPC were only conducted in Chinese [1,4,5,37]. It remains uncertain whether the same carcinogenesis mechanism could apply to different Chinese sub-group or non-Chinese populations.

Our findings highlight the importance of examining the relations between key risk factor exposures and NPC on multiple jurisdictions rather than focusing on a single locality and a single exposure in an ecological study. Furthermore, since NPC includes a spectrum of squamous, and keratinising versus non-keratinising subtypes which are linked to differential risk factors, this may also be an important reason underlying the varying patterns of ecological associations found across different jurisdictions. Thus an ecological study investigates the links between risk and protective factors and different subtypes would be of interest, especially when the pathogenesis processes for various NPC subtypes appear to be distinct. Therefore it is important to first elucidate the secular trend of salted fish consumption and NPC incidence/mortality rates between multiple areas. The findings from this study have encouraged us to further examine the time trend of different NPC subtypes across regions, and so far our literature review has shown that a more thorough review and deeper understanding is needed of different NPC histological classification between China and the West, but such data are often scarce. Also, the differences between Chinese and WHO classifications need to be further examined and resolved. We believe this would warrant a separate paper for a detailed discussion.

There are several limitations in our study. First of all we could not be certain that the capture of NPC incidence and mortality was 100% complete and accurate in each jurisdiction, but most data were derived from cancer registries where completeness of data; especially that of mortality, is good with high accuracy. Variations of diagnostic criteria could exist but this effect is minimised by using International Classification of Disease (ICD 8–10) since NPC is a rare and uncommon cancer. Secondly, per capita consumption was calculated by taking the difference between export, import, production and re-export of salted fish. Consequently, there was a possibility of over or underestimating the actual amount of salted fish being consumed. Nevertheless, data obtained from Hong Kong Trade and Statistics and from the FAO were largely consistent. Even though we tried to obtain all the salted fish related data as precisely as possible, the data we used might include other dried fish products rather than just Cantonese style salted fish, details of which could not be obtained from other jurisdictions. Moreover, the uncertainty of latency period between the consumption patterns and ASIR/ASMR is well acknowledged, for that we included the sensitivity analysis by using year of cumulative consumption for regression and result were shown to be robust. Furthermore, vegetable consumption in Hong Kong was not available from Agriculture, Fisheries and Conservation Department HKSAR prior to 1991, thus multivariable analysis was done only between 1991–2008 but not from 1978–2008.

Finally, the limitations of ecological studies due to the ecological fallacy are well recognised, and unknown, unmeasured or immeasurable confounding factors may be present. We tried to take account of important and known factors by accounting for tobacco consumption, vegetable consumption and migration effects, which is the first example of its kind in NPC epidemiological studies. In future studies, age-specific rather than age-standardised mortality and incidence rates could be more informative to investigate disease risk across different ages and in different populations. Because NPC is endemic mainly in Southern China, ecological analysis focusing on the endemic regions of China would yield more important information than a national study. Data specifically in Southern China on salted fish, vegetable and cigarette consumptions, together with NPC incidence and mortality rates (with subtypes) would be highly valuable if such data are available in the near future and more in depth analysis is warranted.

## Conclusions

In this study, we found markedly decreasing trends of NPC ASIR and ASMR in Hong Kong over the past 3 decades, which was correlated with corresponding secular changes in salted fish consumption per capita. However, this association no longer held after adjustment for tobacco and vegetable consumption per capita. Our result showed vegetable had a protective role in NPC ASIR. There was no clear or consistent pattern in the relations between NPC ASIR and ASMR with salted fish consumption across 7 regions in 3 continents. Whether the pathogenic role of salted fish consumption is specific to NPC among Hong Kong Chinese requires further studies.

## Competing interests

The authors have no conflict of interests.

## Authors’ contributions

HYL and CML conducted data analysis. YHC and CML participated in data collection. YHC helped in sequence alignment. HYL drafted the manuscript. AWML, DLWK, MLL and THL helped to draft the manuscript. All authors read and approved the final manuscript.

## Pre-publication history

The pre-publication history for this paper can be accessed here:

http://www.biomedcentral.com/1471-2407/13/298/prepub

## Supplementary Material

Additional file 1: Table S1 Population in Hong Kong aged 5 and over by usual language, 1991, 1996, 2001 and 2006. **Table S2.** Population by nationality in Hong Kong 1991, 1996, 2001 and 2006. **Table S3.** Population by place of birth in Hong Kong 1961-2006. Click here for file
