# Supplementary material for: Secular trends of salted fish consumption and nasopharyngeal carcinoma: a multi-jurisdiction ecological study in 8 regions from 3 continents
Source: BMC Cancer. 2013 Jun 19;13:298. doi: 10.1186/1471-2407-13-298 (PMC3729410; doi:10.1186/1471-2407-13-298)
Supplement: Additional file 1: Table S1 — Population in Hong Kong aged 5 and over by usual language, 1991, 1996, 2001 and 2006. Table S2. Population by nationality in Hong Kong 1991, 1996, 2001 and 2006. Table S3. Population by place of birth in Hong Kong 1961-2006. [file 1471-2407-13-298-S1.ppt]

## Slide 1
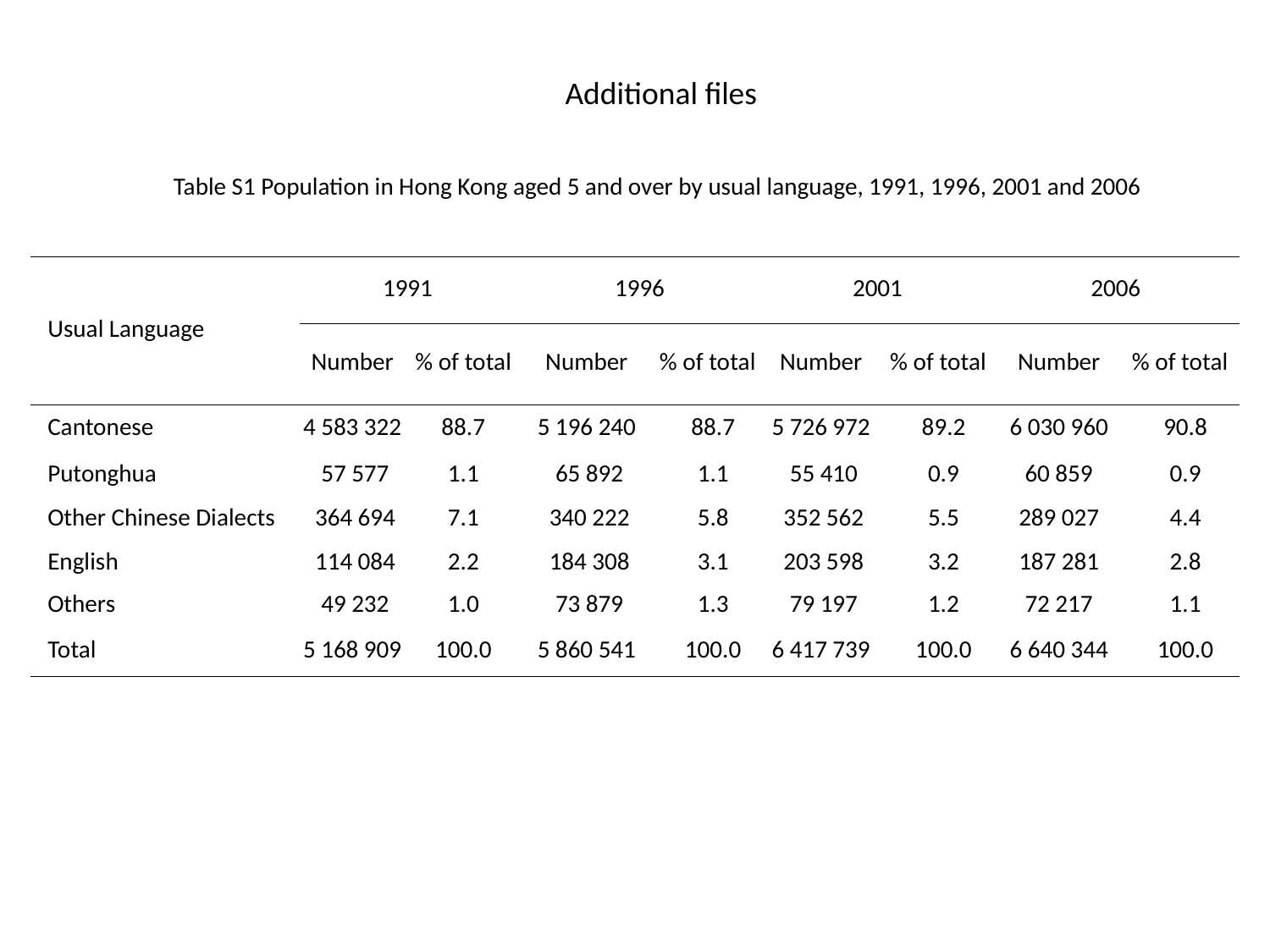

Additional files
Table S1 Population in Hong Kong aged 5 and over by usual language, 1991, 1996, 2001 and 2006
| Usual Language | 1991 | | 1996 | | 2001 | | 2006 | |
| --- | --- | --- | --- | --- | --- | --- | --- | --- |
| | Number | % of total | Number | % of total | Number | % of total | Number | % of total |
| Cantonese | 4 583 322 | 88.7 | 5 196 240 | 88.7 | 5 726 972 | 89.2 | 6 030 960 | 90.8 |
| Putonghua | 57 577 | 1.1 | 65 892 | 1.1 | 55 410 | 0.9 | 60 859 | 0.9 |
| Other Chinese Dialects | 364 694 | 7.1 | 340 222 | 5.8 | 352 562 | 5.5 | 289 027 | 4.4 |
| English | 114 084 | 2.2 | 184 308 | 3.1 | 203 598 | 3.2 | 187 281 | 2.8 |
| Others | 49 232 | 1.0 | 73 879 | 1.3 | 79 197 | 1.2 | 72 217 | 1.1 |
| Total | 5 168 909 | 100.0 | 5 860 541 | 100.0 | 6 417 739 | 100.0 | 6 640 344 | 100.0 |

## Slide 2
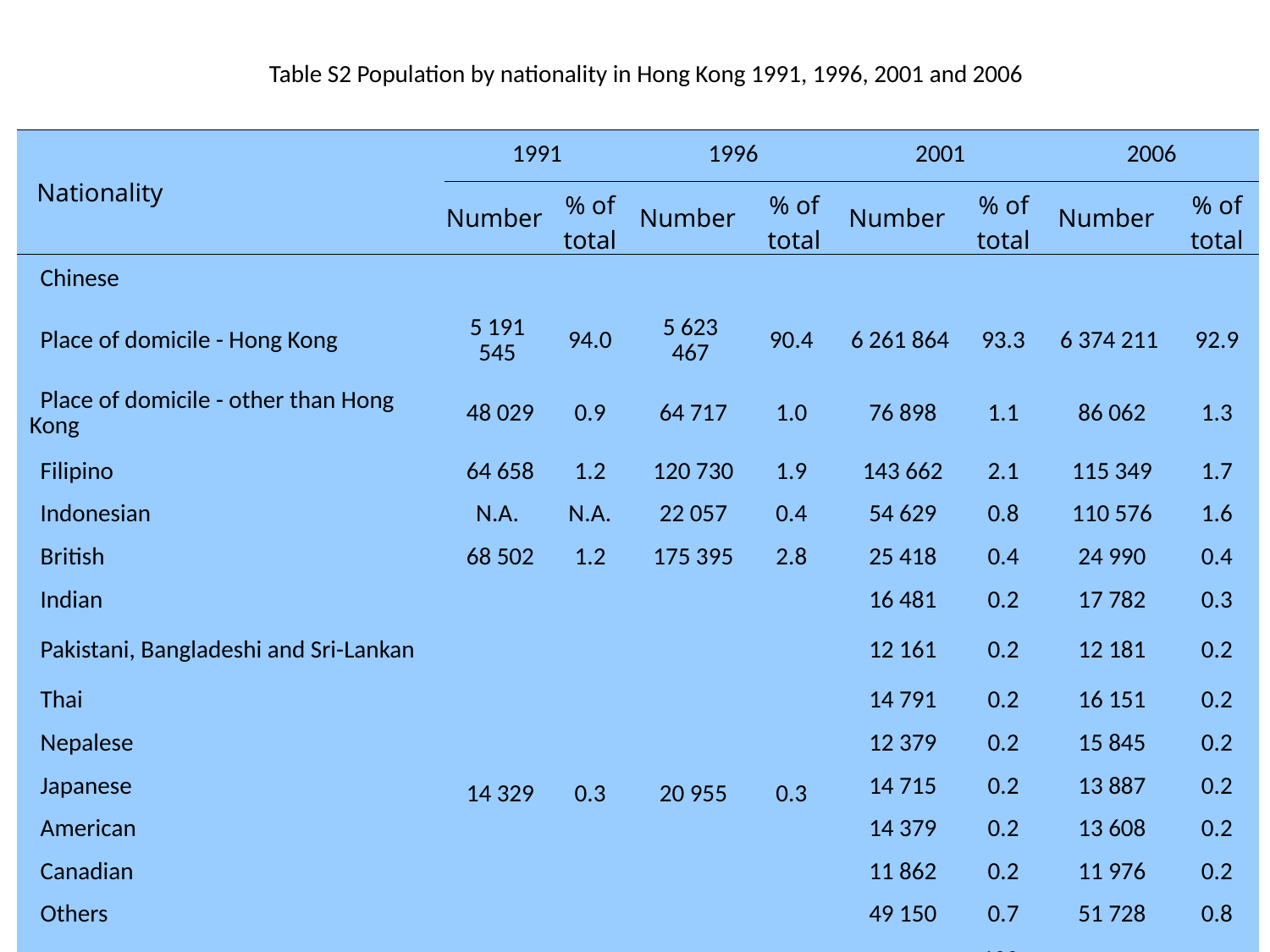

Table S2 Population by nationality in Hong Kong 1991, 1996, 2001 and 2006
| Nationality | 1991 | | 1996 | | 2001 | | 2006 | |
| --- | --- | --- | --- | --- | --- | --- | --- | --- |
| | Number | % of total | Number | % of total | Number | % of total | Number | % of total |
| Chinese | | | | | | | | |
| Place of domicile - Hong Kong | 5 191 545 | 94.0 | 5 623 467 | 90.4 | 6 261 864 | 93.3 | 6 374 211 | 92.9 |
| Place of domicile - other than Hong Kong | 48 029 | 0.9 | 64 717 | 1.0 | 76 898 | 1.1 | 86 062 | 1.3 |
| Filipino | 64 658 | 1.2 | 120 730 | 1.9 | 143 662 | 2.1 | 115 349 | 1.7 |
| Indonesian | N.A. | N.A. | 22 057 | 0.4 | 54 629 | 0.8 | 110 576 | 1.6 |
| British | 68 502 | 1.2 | 175 395 | 2.8 | 25 418 | 0.4 | 24 990 | 0.4 |
| Indian | 14 329 | 0.3 | 20 955 | 0.3 | 16 481 | 0.2 | 17 782 | 0.3 |
| Pakistani, Bangladeshi and Sri-Lankan | | | | | 12 161 | 0.2 | 12 181 | 0.2 |
| Thai | 11 787 | 0.2 | 15 993 | 0.3 | 14 791 | 0.2 | 16 151 | 0.2 |
| Nepalese | N.A. | N.A. | N.A. | N.A. | 12 379 | 0.2 | 15 845 | 0.2 |
| Japanese | 10 850 | 0.2 | 19 010 | 0.3 | 14 715 | 0.2 | 13 887 | 0.2 |
| American | 18 383 | 0.3 | 28 946 | 0.5 | 14 379 | 0.2 | 13 608 | 0.2 |
| Canadian | 15 135 | 0.3 | 32 515 | 0.5 | 11 862 | 0.2 | 11 976 | 0.2 |
| Others | 79 063 | 1.4 | 93 771 | 1.5 | 49 150 | 0.7 | 51 728 | 0.8 |
| Total | 5 522 281 | 100.0 | 6 217 556 | 100.0 | 6 708 389 | 100.0 | 6 864 346 | 100.0 |

## Slide 3
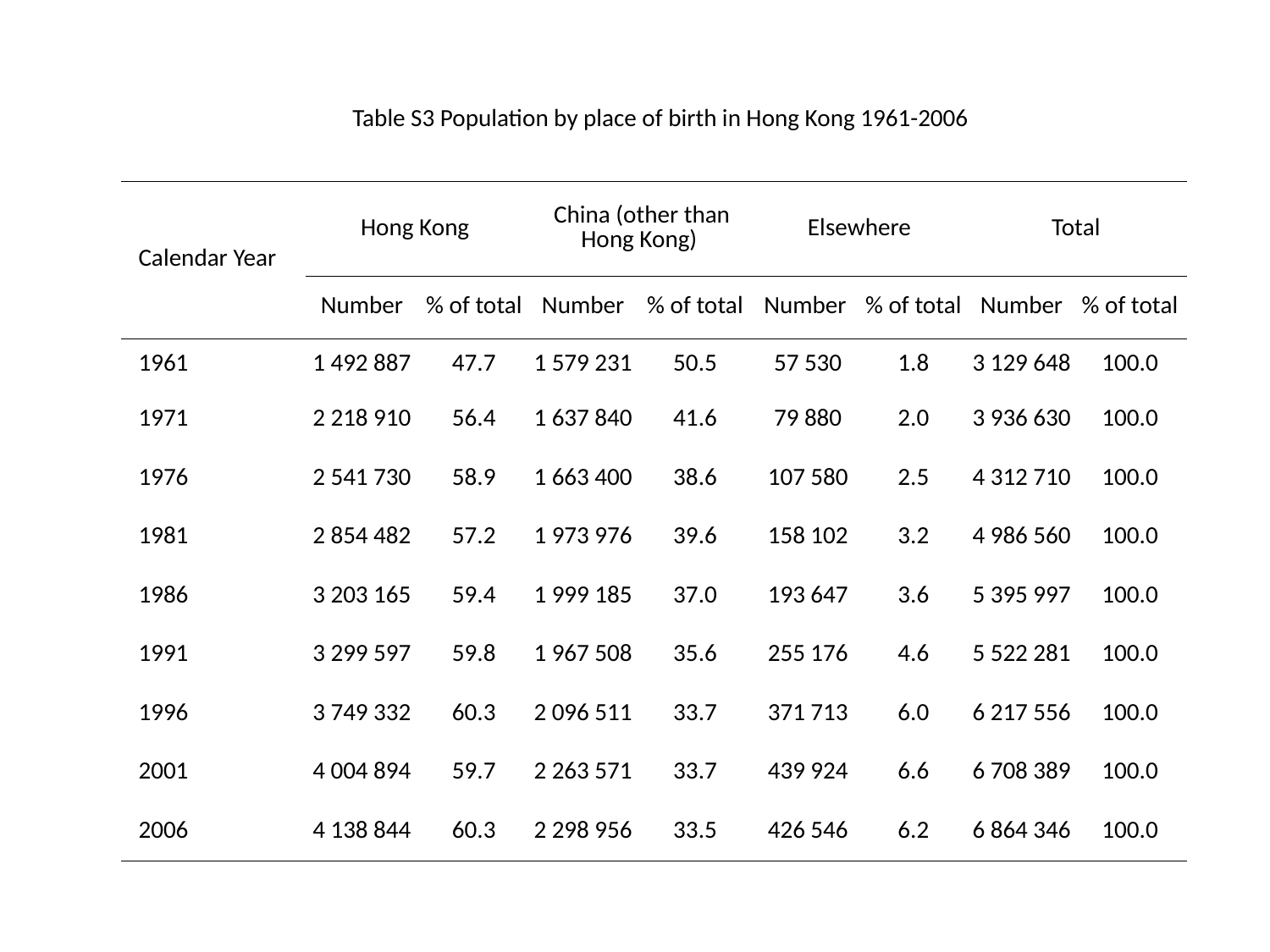

Table S3 Population by place of birth in Hong Kong 1961-2006
| Calendar Year | Hong Kong | | China (other than Hong Kong) | | Elsewhere | | Total | |
| --- | --- | --- | --- | --- | --- | --- | --- | --- |
| | Number | % of total | Number | % of total | Number | % of total | Number | % of total |
| 1961 | 1 492 887 | 47.7 | 1 579 231 | 50.5 | 57 530 | 1.8 | 3 129 648 | 100.0 |
| 1971 | 2 218 910 | 56.4 | 1 637 840 | 41.6 | 79 880 | 2.0 | 3 936 630 | 100.0 |
| 1976 | 2 541 730 | 58.9 | 1 663 400 | 38.6 | 107 580 | 2.5 | 4 312 710 | 100.0 |
| 1981 | 2 854 482 | 57.2 | 1 973 976 | 39.6 | 158 102 | 3.2 | 4 986 560 | 100.0 |
| 1986 | 3 203 165 | 59.4 | 1 999 185 | 37.0 | 193 647 | 3.6 | 5 395 997 | 100.0 |
| 1991 | 3 299 597 | 59.8 | 1 967 508 | 35.6 | 255 176 | 4.6 | 5 522 281 | 100.0 |
| 1996 | 3 749 332 | 60.3 | 2 096 511 | 33.7 | 371 713 | 6.0 | 6 217 556 | 100.0 |
| 2001 | 4 004 894 | 59.7 | 2 263 571 | 33.7 | 439 924 | 6.6 | 6 708 389 | 100.0 |
| 2006 | 4 138 844 | 60.3 | 2 298 956 | 33.5 | 426 546 | 6.2 | 6 864 346 | 100.0 |
